# Chronic unpredictable stress regulates visceral adipocyte‐mediated glucose metabolism and inflammatory circuits in male rats

**DOI:** 10.14814/phy2.284

**Published:** 2014-05-16

**Authors:** Iordanes Karagiannides, Viktoriya Golovatscka, Kyriaki Bakirtzi, Aristea Sideri, Martha Salas, Dimitris Stavrakis, Christos Polytarchou, Dimitrios Iliopoulos, Charalabos Pothoulakis, Sylvie Bradesi

**Affiliations:** 1 Inflammatory Bowel Disease Center, and Neuroendocrine Assay Core, Division of Digestive Diseases, David Geffen School of Medicine at UCLA, Los Angeles, 90095, California; 2 Division of Digestive Diseases, Center for Neurobiology of Stress, CURE VAGLA HC, Los Angeles, 90073, California; 3 Department of Cancer Immunology & AIDS, Dana‐Farber Cancer Institute, Boston, 02215, Massachusetts; 4 90095 Department of Microbiology & Immunobiology, Harvard Medical School, Boston, 02115, Massachusetts; 5 Center for Systems Biomedicine, Division of Digestive Diseases, David Geffen School of Medicine at UCLA, Los Angeles, 90095, California; 6 Division of Digestive Diseases, David Geffen School of Medicine at UCLA, Los Angeles, California

**Keywords:** Adipocytes, adipokines, fat tissue, insulin resistance, stress

## Abstract

Chronic psychological stress is a prominent risk factor involved in the pathogenesis of many complex diseases, including major depression, obesity, and type II diabetes. Visceral adipose tissue is a key endocrine organ involved in the regulation of insulin action and an important component in the development of insulin resistance. Here, we examined for the first time the changes on visceral adipose tissue physiology and on adipocyte‐associated insulin sensitivity and function after chronic unpredictable stress in rats. Male rats were subjected to chronic unpredictable stress for 35 days. Total body and visceral fat was measured. Cytokines and activated intracellular kinase levels were determined using high‐throughput multiplex assays. Adipocyte function was assessed via tritiated glucose uptake assay. Stressed rats showed no weight gain, and their fat/lean mass ratio increased dramatically compared to control animals. Stressed rats had significantly higher mesenteric fat content and epididymal fat pad weight and demonstrated reduced serum glucose clearing capacity following glucose challenge. Alterations in fat depot size were mainly due to changes in adipocyte numbers and not size. High‐throughput molecular screening in adipocytes isolated from stressed rats revealed activation of intracellular inflammatory, glucose metabolism, and MAPK networks compared to controls, as well as significantly reduced glucose uptake capacity in response to insulin stimulation. Our study identifies the adipocyte as a key regulator of the effects of chronic stress on insulin resistance, and glucose metabolism, with important ramifications in the pathophysiology of several stress‐related disease states.

## Abstract (precis)

e00284

Our study identifies the adipocyte as a key regulator of the effects of chronic stress on insulin resistance, and glucose metabolism, with important ramifications in the pathophysiology of several stress‐related disease states.

## Introduction

The epidemic spread of metabolic disorders associated with obesity and the severe complications involved (type II diabetes, insulin resistance, cardiovascular diseases, gastrointestinal disorders, and symptoms) pose a major challenge to public health and modern medicine. The prevalence of obesity and metabolic syndrome in the US is 33% and 24%, respectively, and is rapidly rising, creating challenges for an already burdened health system (Bray and Bellanger 2006; Ogden et al. 2010). Increasing evidence shows an association between chronic psychological stress or depression and type 2 diabetes, visceral obesity, dyslipidemia, and impaired glucose tolerance, significantly compromising quality of life and life expectancy (Bose et al. 2009; van Reedt Dortland 2013). Although the epidemiological link between chronic stress, visceral fat accumulation, and increased risks of metabolic disorders is striking (Speaker and Fleshner 2012), the precise biological mechanisms underlying the effect of stress on visceral adipose tissue and in particular adipocyte function, and the adverse impact of visceral fat in stress‐related conditions have yet to be established.

With the emergence of adipose tissue as a major endocrine organ (Ahima and Flier 2000; Fortuno et al. 2003), numerous reports link changes in adipocyte physiology and function with common life‐threatening pathological conditions, including inflammatory bowel disease (IBD), hypertension, and cancer (Shively et al. 2009). Moreover, insulin resistance and the subsequent development of non‐insulin‐dependent (type II) diabetes mellitus (NIDDM) are associated with a low‐level inflammatory state and altered fat cell function during chronic obesity (Hotamisligil 2010). Indeed, several adipocyte‐derived molecules (adipokines) including TNF*α*, IL‐6, and adiponectin, among others, have the capacity either to promote or inhibit insulin signaling (Marra and Bertolani 2009).

A proposed mechanism for the impairment of insulin signaling by cytokines and fatty acids involves the inhibitory serine phosphorylation of insulin receptor substrate‐1 (IRS‐1), an insulin receptor docking protein essential for downstream signaling (Morino et al. 2006; Tanti and Jager 2009). The mechanism of this response involves several stress‐activated kinases such as c‐Jun N‐terminal kinase (JNK), protein kinase C (PKC), and the inhibitor of nuclear factor‐*κ*B kinase (IKK) (Hirosumi et al. 2002; Kim et al. 2004). JNK is activated by extracellular signals such as cytokines and fatty acids and its importance on insulin resistance is demonstrated in JNK knock‐out mice which maintain normal insulin signaling function IRS‐1 serine phosphorylation during obesity (Hotamisligil 2006). Extracellular fatty acids also activate PKC*θ*, while PKC*θ*‐deficient mice are protected against insulin resistance (Hotamisligil 2006). Moreover, the effects of PKC*θ* on insulin signaling are mediated through activation of IKK, a kinase that promotes insulin resistance (Yuan et al. 2001).

The role of inflammation in the promotion of insulin resistance is also highlighted by evidence suggesting a central role of MAPKs and the IKK*β*/NF‐kB pathway in the inhibition of insulin signaling in different tissues. The importance of ERK1 activation was demonstrated by mouse models lacking either ERK1 or its inhibitor, the signaling adaptor p62 (Bost et al. 2005; Rodriguez et al. 2006). In muscle, inhibition of p38 MAPK improved insulin‐stimulated glucose transport via the prevention of IRS‐1 degradation (Archuleta et al. 2009). In a similar manner, the IKK*β*/NF‐*κ*B promotes insulin resistance in hepatocytes and hypothalamus (Cai et al. 2005; Zhang et al. 2008).

Stress is defined as a threat to the homeostasis of an organism and chronic exposure with sustained hyperactivity of the endocrine stress system leads to various pathological states. Visceral adipose tissue (VAT) and HPA axis are known to directly influence each other, and inflammatory adipokines from the VAT activate the HPA axis (Kyrou et al. 1083). In humans, women are more sensitive to the obesity‐promoting effects of psychological stress, while men have a higher risk of accumulating fat in visceral depots (Bjorntorp 1996; Razzouk and Muntner 2009). Studies in rats demonstrated higher activity of the enzyme 11b‐hydroxysteroid dehydrogenase (11b‐HSD1, which generates active glucocorticoid (GC) from inactive GC metabolites within tissues) in visceral versus subcutaneous adipose tissue in males, which may contribute to increased visceral adiposity in males during chronic stress (Nieuwenhuizen and Rutters 2008). An alternative contributing mechanism to the effect of stress on adiposity includes activation of the autonomic nervous system and differential engagement of CNS polysynaptic projections to adipose tissues during chronic stress, which may influence fat distribution patterns (Bartolomucci and Leopardi 2009; Adler et al. 2012). Furthermore, impaired glucose tolerance is associated with visceral fat accumulation (Lamounier‐Zepter et al. 2006; Bose et al. 2009). In adipocytes, glucocorticoids regulate differentiation and proliferation as well as the activation of insulin signaling pathways (Buren et al. 2008). While pro‐inflammatory mediators are secreted by adipose tissue and their secretion is influenced by increased adiposity (Perrini et al. 2008), the mechanisms by which stress contributes to general inflammatory states associated with increased visceral adiposity remain unknown.

In this study, we employed the chronic unpredictable stress (CUS) rat model that leads to anxiety features comparable to humans and validated in several published reports as a well‐characterized model of depression symptoms with high predictive validity (Barsy et al. 2010; Hawley et al. 2010). Our data demonstrate significant alterations in the lean/fat mass ratios and changes in intraabdominal fat depot sizes with chronic stress. These changes in body composition are accompanied by impaired glucose uptake capacity in response to insulin in adipocytes following stress. As expected, these changes were associated with activation of intracellular inflammatory and proliferative circuits in mesenteric and epididymal adipocytes. These are the first direct results pointing to the adipocyte as an important contributor as well as a target cell type involved in stress‐induced effects on glucose‐associated inflammatory and metabolic responses.

## Materials and Methods

### Animals

Male Fisher rats ([225‐250 g], ~12 weeks old) were purchased from Harlan, IN. We used groups of *n *=**6‐12 for each study. Rats were maintained on a normal light–dark cycle, and provided with food and water ad libitum, with variations according to the chronic stress paradigm. All protocols were approved by the Institutional Animal Care and Use Committee at the VA Greater Los Angeles Healthcare System and all animal experiments were carried out in accordance with the National Institute of Health Guide for the Care and Use of Laboratory Animals (NIH Publications No. 80 23, revised 1978).

### Echo MRI measurement of total body fat/lean mass

The EchoMRI (Echo Medical Systems, Houston, TX) is a system based on Nuclear Magnetic resonance which provides the best accuracy for the measurement of body composition. The measurement chamber is specifically designed for rats to not induce injuries or lesions. The rats were placed awake in the chamber for about 2 min and removed from the chamber and placed back in their home cages as soon as the measurement was finished.

### Collection of visceral fat samples

For mesenteric fat depot EchoMRI measurements, the intestine was removed from the anus to the stomach and composition was determined after detachment from the pancreas and removal of the cecum (without the attached fat).

### Adipocyte size measurement

Paraffin‐embedded sections of adipose tissue were placed on slides and stained with H&E. Adipocyte area was then measured under light microscopy (Imager. Z 1, ZEISS, Chester, VA) using the AxioVision (Rel. 4.6) software program. Cell size was determined by outlining the adipocyte circumference (100 cells per rat), while for the determination of cells/area, 10 different and comparable in size adipose tissue areas (per slide) were highlighted and the adipocytes included were counted.

### In vivo blood glucose measurement after glucose and insulin challenge

Chronic unpredictable stress and control rats were placed in restraint tubes for habituation for 30 min/day for 3 days before the experiment. Rats from all groups were fasted overnight and tested for glucose tolerance. Dextrose was then injected i.p. (1 g/kg) and blood samples were drawn at 0, 15, 30, 120, and 240 min post injection. Serum glucose levels were measured using standard glucose test strips. Twenty‐four hours after the glucose challenge experiment, the animals were fasted again and the insulin tolerance test was performed. Insulin (2 Units/kg) was injected i.p. and serum glucose was measured at the same time points as before. Blood collection was performed via the tail vein in rats placed in restraint tubes. These experiments were performed 24 h–72 h after the last stressor.

### In vivo measurement of circulating insulin and NEFA

Insulin and NEFA we measured using the HR Series NEFA kit (WAKO diagnostics, Richmond, VA) in serum samples collected from control and CUS rats at the end of the stressor. In addition, we performed additional measurement of NEFA in response to insulin stimulation (2 Units/kg), 30 min after injection, in CUS rats fasted for 4 h.

### 3H‐2‐deoxy‐d‐glucose uptake

Primary Fisher 344 (*n *=**6) rat adipocytes from epididymal fat depots were isolated as described (Karagiannides et al. 2001), placed in 1.5‐mL Eppendorf tubes, and transferred to serum‐free DMEM 4 h before the experiments. Experiments were performed as described in Karagiannides et al. (2011). Briefly, cells were washed with glucose‐free KRH medium (121 mm NaCl, 4.9 mm KCl, 1.2 mm MgSO_4_, 0.33 mm CaCl_2_, and 12 mm HEPES acid [pH 7.4]) and treated with 100 nm insulin (Sigma, St. Louis, MO) for 15 min. ^3^H‐2‐deoxy‐d‐glucose/2‐deoxy‐d‐glucose cocktail (specific activity, 6.25 mCi/mmol) was added for 3.5 min at 37 C. Glucose uptake was stopped by washing the cells with ice‐cold KRH with 25 mm glucose and 10 *μ*m cytochalasin B for three times. Cells were then collected in glucose‐free KRH with 0.1% sodium dodecyl sulfate and were subjected to liquid scintillation counting in EcoLume (ICN Biomedicals, Costa Mesa, CA). Radioactive glucose levels were corrected for the total adipocyte protein content per tube.

### Multiplex cytokine and phosphoprotein immunoassays

Cytokine concentrations in rat serum and adipose tissue were determined using the Bio‐Plex Pro Rat Cytokine 24‐plex Assay multiplex immunoassays (Bio‐Rad, Hercules, CA) and the final data were obtained and analyzed via the Bio‐plex 3D Suspension array system (Bio‐Rad). For the determination of intracellular kinases activation levels we used a combination of Bio‐plex nonmagnetic signal transduction assays (Bio‐Rad). Loading for the phosphoprotein panels as well as the adipose tissue cytokine panels was normalized via determination of total protein concentration (BCA Protein Assay kit, Pierce, Rockford, IL) and measurement of *β*‐actin levels after western immunoblot analysis.

### Real‐time PCR

1 *μ*g of RNA isolated was reverse transcribed into cDNA and incubated with dual fluorogenic probes (Applied Biosystems, Foster City, CA). GAPDH and 18s were used as endogenous controls and were also detected using dual‐labeled fluorogenic probe (5′‐FAM/3′‐MGB probe, Applied Biosystems). Target mRNA (TNF*α*, IL‐6, MCP‐1, and IL‐1*β*, Applied Biosystems) levels were quantified using a fluorogenic 5′‐nuclease PCR assay as described in Karagiannides et al. (2011) using a 7500 Fast Real‐Time PCR sequence detection system (Applied Biosystems).

### Expression microarrays and analysis

RNA for microarray analysis was isolated from rat epididymal adipocytes using the RNeasy mini kit (QIAGEN, Valencia, CA). Gene expression profiling was performed at the UCLA Clinical Microarray Core using the Agilent SurePrint G3 Rat Exon Microarray platform. Differentially expressed genes were generated using the dChip software program.

Using these differentially expressed gene data, gene networks were constructed and identified important hubs using Ingenuity Gene Network Analysis. Pathways of highly interconnected genes were identified by statistical likelihood using the following equation:
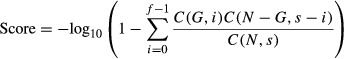


Where *N* is the number of genes in the network of which *G* are central node genes, for a pathway of *s* genes of which *f* are central node genes. C is the binomial coefficient. We considered statistically significant networks those with a score greater than 5 (*P *< 10^−5^). Central node genes were considered the ones known to be regulated by or to regulate the highest number of genes shown in our study to change with stress. The calculation of the significance of these networks along with the background gene–gene interaction analysis was performed by the IPA program of Ingenuity systems.

The data discussed in this publication have been deposited in NCBI's Gene Expression Omnibus (Edgar et al. 2002) and are accessible through GEO Series accession number GSE47754. http://www.ncbi.nlm.nih.gov/geo/query/acc.cgi?acc=GSE47754

### Chronic unpredictable stress

Rats are exposed to a variable sequence of stressors, 2 per day, for 35 days. The stressors onset was applied at random time during the day between 10 am and 2 pm and overnight stressors started at random time between 4 and 7 pm. This stress model has been validated in numerous reports and is a well‐characterized model of depression in human with high face and predictive validity (Willner et al. 1987; Barsy et al. 2010; Hawley et al. 2010; Pechlivanova et al. 2010). Briefly, stressors were applied based on a computer‐generated randomized sequence. All animals were housed in pairs and then single housed during isolation stress. Control animals were handled every 2 days. An additional control group was added to test the effect of a single day of stress. Groups of rats were exposed to crowding and strobe light overnight (*n *=**9) or handled and left in their home cage and housing conditions as controls (*n *=**8).

### Schedule of stressors 2/day for 35 days (computer randomized sequence)

Swim stress 18°C 10 min (between 10 am and 2 pm): Days 25, 27, 29, 30, 32, 33, 34
Rotation 1 h (between 10 am and 2 pm): Days 1, 2, 4, 6, 9, 11, 14, 20, 31
Isolation overnight: Days 7, 12
Food/water deprivation overnight: Days 2, 3, 6, 10, 13, 15, 16, 19, 24, 28, 32
Light on overnight: Days 1, 4, 7, 8, 13, 18, 22, 23
Light off 3 h (between 10 am and 2 pm): Days 5, 8, 11, 20, 21, 24, 26, 28, 31
Stroboscope overnight: Days 5, 15, 16, 17, 19, 21, 26, 27, 29, 33, 34, 35
Wet bedding overnight: Days 3, 12, 14,
Crowding overnight: Days 9, 10, 17, 18, 22, 23, 25, 30, 35

#### Stressor details

*Swim stress 18°C 10 min (between 10 am* *and 2 pm)*: Rats are placed for 10 min in a cylindrical clear plastic tank (46 cm high × 20 cm diameter) filled with water (18 ± 1°C) to a depth of 30 cm. The rats are closely monitored during this stress and should any rat fail to show the reflex swimming behavior, it will be removed from the water to prevent drowning. Immediately after the swim, rats are removed from the tank, towel dried, and put in a warming cage (37°C) for 5 min before being placed back in home cage. This procedure, allowing the rat to dry before being placed in his home cage, prevents chills from the wet environment.

*Rotation 1 h (between 10 am* *and 2 pm)*: The cage was placed on a 120 rpm rocking bed. During this stress the rat is placed in a cage positioned on a rotating device.

*Isolation overnight:* single housing in a separate room.

*Food/water deprivation overnight:* This procedure is routinely used in various animal studies and does not cause physical harm to the animal. Food and water are provided immediately after the end of the fasting period.

*Light on overnight*: Light switch is kept on for 24 consecutive hours.

*Light off 3 h (between 10 am* *and 2 pm)*: Light is switched off at random time during the day for 3 h.

*Stroboscope overnight*: Low intensity stroboscopic ‐illumination (300 flashes/min).

*Wet bedding overnight:* Damp bedding (200‐mL water in a cage). Immediately after the stress, rats are removed from the cage, towel dried, and put in a warming cage (37°C) for 5 min before being placed back in home cage as described above.

*Crowding overnight:* Five rats cohabiting in one small cage (425 × 265 × 180 mm). Rats were examined carefully during and after each session for signs of injury or sores. Dominant rats were identified and were replaced.

### Statistical analysis

Results were analyzed using the Prism professional statistics software program (Graphpad Software Inc., San Diego, CA). ANOVA and nonparametric tests with unequal variance were used to assess the statistical difference between controls and CUS. *P *<**0.05 was considered significant. For the detection of differentially expressed genes in adipocytes using dChip we used unpaired t‐test with a twofold expression difference and *P *<**0.05 cutoffs.

## Results

### Chronic stress alters body and fat depot weight in rats

**Figure 1.**
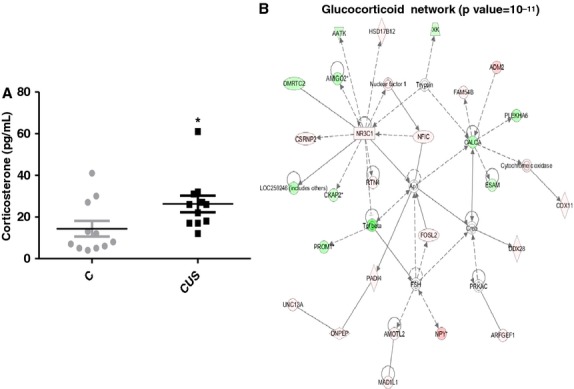
Chronic stress induces increase in corticosterone levels and related responses. (A) ELISA in plasma isolated from blood of stressed and control rats shows that corticosterone levels increase with stress while (B) the glucocorticoid network is also significantly affected with NR3C1 (receptor for glucocorticoid) representing a central node in the network. Data are means ± SEM (Mann–Whitney, *P <**0.05, *n *= 11).

**Figure 2.**
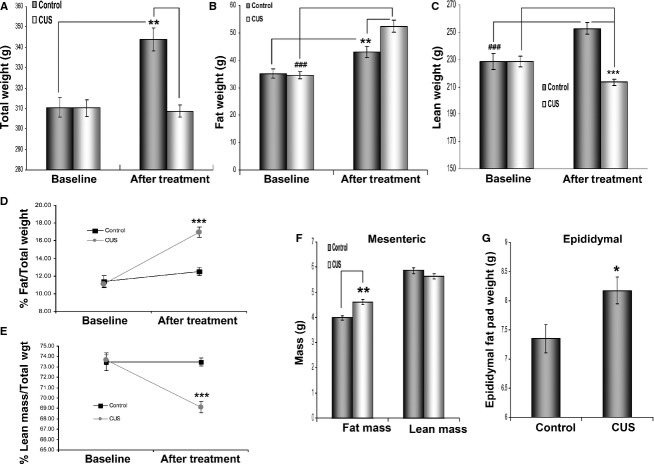
Stress‐induced effects on rat weight, body composition, and fat depot size. Male rats were weight matched and placed in two groups (*n *=**6/group). The CUS group was subjected to different combinations of stressors for 35 days while the control group was caged under normal conditions. (A) Weight comparison between stressed and control rats before and after treatment shows that while animals in the control group gain weight after 35 days, the weight of rats in the chronic stress group remains unaffected after completion of the stress protocol. (B) However, stressed rats gain significantly more total body fat in comparison to their control littermates. (C) The lack of total weight gain in the stressed rats is due to the reduction in total lean mass in these animals in contrast to the control rats that also show increases in lean mass as in fat mass before. (D) EchoMRI analysis demonstrates that, in rats, % fat mass increases considerably (~6%) relative to the rest of body mass after stress (E) while% lean mass decreases (~4.5%). (F) Comparison of lean and mesenteric fat mass of the intestine between stressed and control rats shows that the former have higher fat mass weight while no significant difference exists in lean intestinal mass between the two groups. (G) Stressed rats also have increased epididymal fat pad weights compared to control rats after 35 days of stress. Data are means ± SEM (Mann–Whitney, *P <**0.05, **P <**0.01, *** and ^###^*P *<**0.001, *n *=**6 in four independent studies).

The effect of stress at the molecular level was assessed at the end of the experiment by measuring circulating corticosterone levels. We found that CUS induced an almost twofold increase in corticosterone levels in the blood (Fig. 1A, 14.36 vs. 26.27 pg/mL, *P *=**0.03, *n *=**11) of stressed rats. Consistent with the expression data, gene network analysis showed a significant (*P *= 10^−11^) activation of a glucocorticoid gene network, having as central node of the network the glucocorticoid receptor (NR3C1), which is highly upregulated (Fig. 1B). Chronically stressed rats failed to gain weight during the stress period (Fig. 2A, 308 ± 3 g vs. 310 ± 4, *n *=**12) compared to controls, which showed a physiological increase in total body weight (Fig. 2A, 343.8 ± 5.6 vs. 310.4 ± 4.7, *P *<**0.01, *n *=**12). Further analysis using echoMRI showed that the lack of weight gain in stressed rats may be attributed to loss of lean mass (Fig. 2C, *P *<**0.001, *n *=**12), while the overall fat mass of these animals increased significantly (Fig. 2B, stress *P *<**0.001, control *P *<**0.01, *n *=**12).

Overall, stressed rats gained significantly more fat compared to nonstressed controls (Fig. 2D, 5.8 ± 0.6 vs. 1.1 ± 0.7, as % of total body weight, respectively, *P *<**0.001). In control animals both fat and lean mass increased accordingly to reflect the overall change in body weight (Fig. 2D and E). In a different cohort, rats exposed to an alternative CUS protocol (without food/water deprivation) showed similar but not statistically significant changes in visceral adiposity (data not shown). In the control group exposed to a single day of stress, there was no change in weight gain between controls and CUS rats, and the% of fat and lean mass to the total body weight was not affected (Data not shown). Interestingly, we did not observe any significant changes on body weight or lean versus fat mass when the model of chronic water avoidance (WA) (1 hr daily for 10 consecutive days) was employed (data not shown) (Bradesi et al. 2009). This may be due to the shorter duration of stress exposure and/or the different nature of the stress procedure.

### Stress increases Intraabdominal fat depot size

Echo MRI analysis did not reveal changes in the amount of intestinal lean mass while mesenteric fat mass increased with stress (Fig. 2F, *P *<**0.01, *n *=**12). The ratio of the fat/lean mass in the mesentery was also higher in stressed rats compared to controls (data not shown, *P *<**0.01, *n *=**12, t‐test). In addition, epididymal fat pads were also removed and weighed at the end of the stress period. Epididymal fat pad weight was significantly higher in stressed rats compared to controls (Fig. 2G, *P *<**0.05, *n *=**12).

### Effect of chronic unpredictable stress on food intake

**Figure 3.**
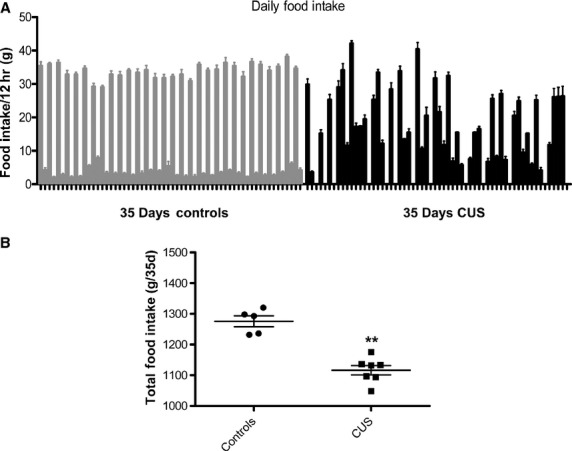
Effects of CUS on rat feeding patterns and overall consumption. Rats undergoing chronic stress were single housed and the food consumption was monitored daily. (A) Daily feeding measurements reveal that rats exhibited irregular feeding patterns compared to controls. Feeding behavior became random irrespective of time of day. (B) Overall food consumption was also reduced in stressed compared to control rats (*P *<**0.01, *n *=**6).

Food intake was measured in gram per 12‐h period. We observed an altered feeding pattern with notable increase in night feedings following exposures to food deprivation overnight (Fig. 3A). However, the total amount of food consumed by CUS rats over the course of the 35 days was significantly lower compared to control rats (Fig. 3B, 1052 ± 66 vs. 1276.18, *P *=**0.02).

### Stress induces changes in fat cell size

**Figure 4.**
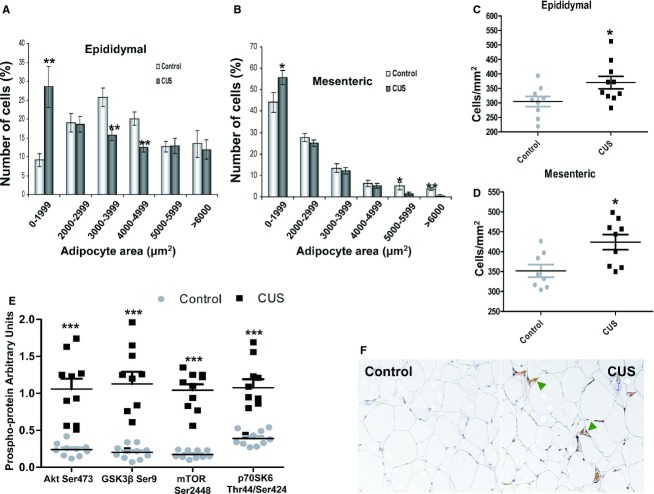
Changes in adipocyte physiology with stress. Paraffin‐embedded mesenteric and epididymal adipose tissue from stressed and control rats was sectioned and stained with H & E. Adipocyte area was then measured under light microscopy and (A,B) an increase in the number of small adipocytes in stressed rats compared to controls. In addition, adipocyte number per section area was calculated and (C) stress led to increases in epididymal as well as (D) in mesenteric adipocytes numbers. Total protein was collected from adipocytes isolated from epididymal fat depots of stressed and control rats and subjected to (E) multiplex phosphoprotein analysis that showed activation of intracellular survival signaling pathways (Akt, GSK3*β*, mTOR, and p70S6K) in epididymal adipocytes. (F) Immunohistochemistry for F4/80 (green arrowheads) shows increased macrophage infiltration in mesenteric adipose tissue of stressed rats. Data are means ± SEM (Mann–Whitney, *P <**0.05, **P <**0.01, ***P <**0.001, *n *=**10).

Epididymal and mesenteric fat depots from stressed animals contained higher percentage of small adipocytes compared to controls (Fig. 4A, epididymal, *P *<**0.01, *n *=**9‐10**;** 4B, mesenteric, *P *<**0.05, *n *=**9‐10). In agreement with increased adiposity shown above, stressed animals also demonstrated increased cell counts per mm^2^ compared to controls (Fig. 4C and D, *P *<**0.05, *n *=**9‐10). Multiplex phosphoprotein analysis revealed increased activating phosphorylation of Akt, mTOR and p70S6K (Fig. 4E, *P *<**0.001, *n *=**10) as well as phosphorylation of inhibitory GSK3*β* (Fig. 4E, *P *<**0.001, *n *=**10) suggesting activation of pro‐survival, antiapoptotic circuits during stress in adipocytes. Immunohistochemistry using an antibody against F4/80 showed increased infiltration of macrophages (green arrowheads) into mesenteric fat depots with stress (Fig. 4F).

### Stress alters glucose homeostasis

**Figure 5.**
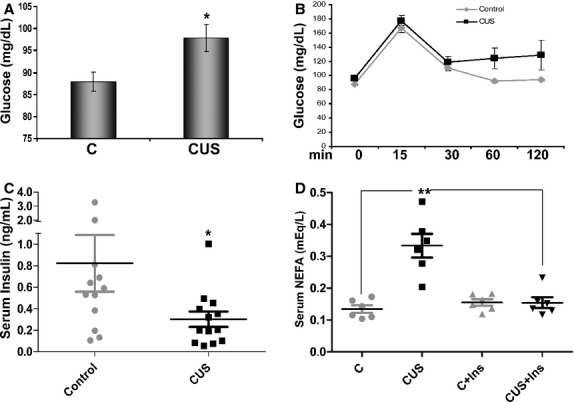
Stress‐induced effects on glucose and NEFA circulating levels. Plasma from stressed and control rats was isolated from whole blood after centrifugation. (A) Chronic stress is associated with increased circulating glucose levels. (D) NEFA plasma levels are elevated in stressed rats compared to controls. At the conclusion of the 35‐day stress protocol, rats were restrained and blood was collected from their tails at the described intervals and measured using an AccuCheck glucose counter. (B) Stressed rats show reduced ability to remove glucose from the circulation after a 2‐hr challenge (glucose 1 g/kg). Circulating glucose levels remain high 60 min after challenge in the stressed group (grey line). (C) Circulating insulin levels decreased with stressed compared to control rats. Data are presented as means ± SEM (Mann–Whitney, One way ANOVA, *P <**0.05, **P <**0.01, *n *=**12 and *n *=**6 for [D]).

Fasting glucose levels were higher in stressed rats compared to controls (Fig. 5A, *P *<**0.05, *n *=**6). In addition, control rats responded more efficiently to glucose challenge compared to stressed rats. Indeed, in control rats, glucose levels were restored to baseline within 1 h after injection, while glucose levels in stressed rats remained significantly higher after 60 min and for the duration of the experiment (Fig. 5B, *P *<**0.05 after 60 min, *n *=**9‐10). In a different cohort, rats were challenged with a higher glucose dose (2 g/kg) and in a similar maker, animals in the control group responded more efficiently to the challenge compared to stressed ones. However, the pattern of the response differed with the most significant responses observed earlier (15 min, *P *<**0.05, *n *=**6) time points of the challenge protocol (Controls 74 ± 1.5, 167.5 ± 15.4, 208.2 ± 27.5, 172.2 ± 21.4, 114 ± 17.6 vs. CUS 92 ± 5, 308.2 ± 51.7, 283.3 ± 51.1, 221.5 ± 21.11, 105.5 ± 7.05 for 0, 15, 30, 60, and 120 min, respectively). In contrast, no changes in response to i.p. insulin administration were observed between the two groups (Data not shown). In the control group exposed to a single (same as the last) day of stress, there was no change in the response to glucose challenge or insulin challenge compared with controls (Data not shown).

### Stress increases plasma nonesterified fatty acid levels and decreases circulating insulin

Elevations in the levels of plasma fatty acids represent a major mechanism of fat‐induced insulin resistance in fat, liver, and muscle tissues via induction of intracellular signaling cascades including activation of Ser/Thr kinases (Morino et al. 2006). When we compared the plasma levels of NEFA between stressed and control rats we observed a significant increase after stress (Fig. 5D, CUS vs. C, *n *=**6, *P *<**0.01). Increased lypolytic activity with stress is also suggested by our expression microarray network analysis that showed significantly increased activation of lipid metabolism networks (*P*=10^‐21^, data not shown, *n *=**6). Circulating insulin levels were significantly lower in stressed rats compared with controls (Fig. 5C, *n *=**12‐13, *P *<**0.05) potentially contributing to the elevated NEFA levels observed after stress via reduced antilipolytic activity in these animals. The levels of circulating NEFA were significantly decreased 30 min after a bolus injection of insulin (Fig. 5D, CUS+Ins, *P *<**0.01) compared to noninjected stressed rats.

### Stress‐induced alterations in the transcriptome of adipocytes in rats

**Figure 6.**
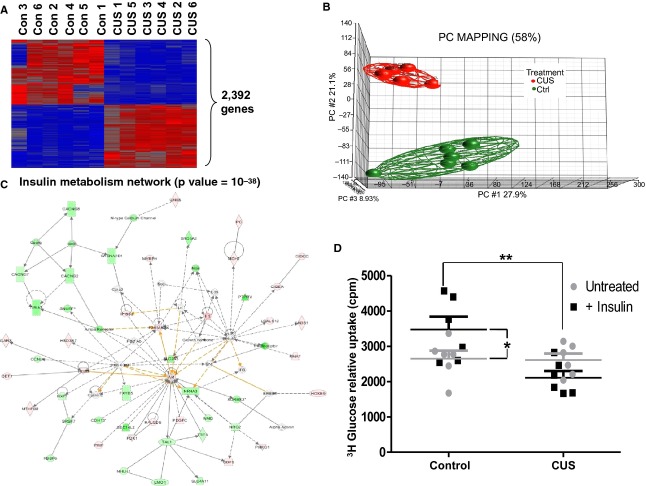
Effects of chronic stress in adipocytes gene expression and response to insulin. Freshly isolated epididymal adipocytes were either placed in Trizol reagent for RNA isolation in 1.5‐mL tubes for 4 h, exposed to insulin and their ability to internalize radioactive (^3^H) glucose is monitored. Insulin stimulates glucose uptake in rat epididymal adipocytes (left panel, black squares, *n *=**6). (A) Stress induces differential expression of 2,392 genes in adipocytes. (B) Primary component mapping showing segregation of differentially expressed genes between adipocytes from stressed and control rats. (C) Network analysis reveals insulin metabolism as the top statistically significant network affected by stress. (D) Stress abolishes the ability of adipocytes to internalize glucose in response to insulin stimulation (right panel, black squares). Data are Mean values ±SEM (Mann–Whitney test, *P <**0.05, **P <**0.01, *n *=**6).

Microarray analysis of the transcriptome of epididymal adipocytes revealed dramatic changes in the expression of over 2,392 genes (Fig. 6A, Differentially expressed genes between 6 CUS and 6 Ctrl at ≥2‐fold, FDR corrected, *P *<**0.05). Primary component (PCA) analysis reveals sharp segregation between the two groups (Fig. 6B). To identify the molecular circuits that are affected in the stress‐induced adipocytes, we integrated the differentially expressed genes into networks. This analysis revealed that the top statistically significant (*P *= 10^−38^) network is related to insulin metabolism, having as central regulators, insulin, AKT kinase, and p38 MAP kinase (Fig. 6C).

### Stress decreases glucose uptake in response to insulin in isolated adipocytes

Adipocytes were isolated at the end of the stress period from control and CUS animals and their ability to internalize radioactive glucose in response to insulin stimulation was measured. In contrast to adipocytes isolated from control rats (Fig. 6D, left), adipocytes isolated from stressed animals failed to internalize glucose in response to insulin stimulation (Fig. 6D, *P <**0.05 and **P <**0.01, *n *=**7).

### Stress increases proinflammatory cytokine levels in rat epididymal fat depots and the circulation

**Figure 7.**
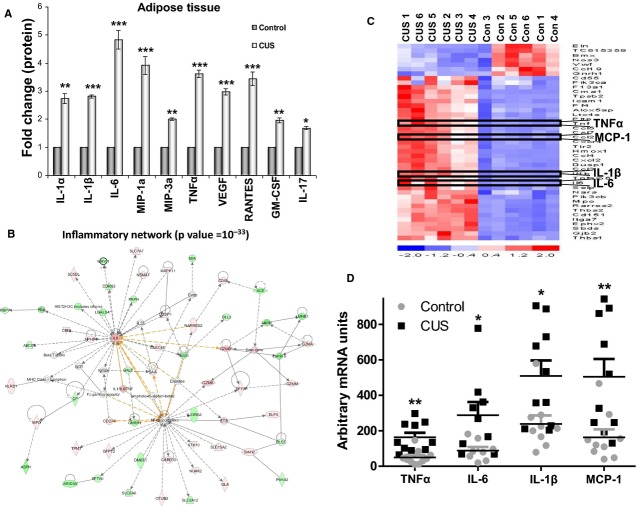
Stress induces inflammatory responses in epididymal fat depots and adipocytes. Epididymal fat depots were removed after stress and were either placed in collagenase for adipocyte isolation or grinded for isolation of total protein to be analyzed via multiplex immunoassay. (A) Proinflammatory cytokine expression is increased in protein extracts from epididymal fat depots from stressed rats compared to controls. We observed increased levels of IL‐1*α*, MIP‐3a, RANTES, GM‐CSF, and IL‐17. (B) Our differential gene expression analysis revealed that the inflammatory network (with IL‐6 as a central node) was significantly affected by stress and (C) four of the genes identified in the heat map of inflammatory genes that increased with stress were TNF*α*, IL‐6, IL‐1*β*, and MCP‐1 and their expression was also verified using (D) real‐time PCR analysis. Data are expressed as means ± SEM (Mann–Whitney, *P <**0.05, **P <**0.01, *n *=**10).

**Figure 8.**
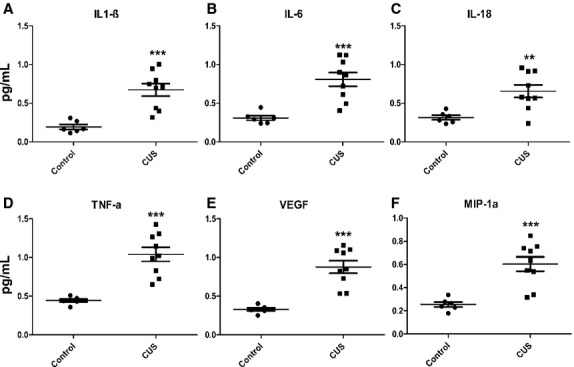
Chronic stress affects the levels of proinflammatory cytokines in the circulation. Blood was collected from stressed and control rats and plasma was isolated after centrifugation. Magnetic bead‐based multiplex immunoassay reveals that the levels of (A) IL‐1*β*, (B) IL‐6, (C) IL‐18, (D) TNF*α*, (E) VEGF, and (F) MIP‐1*α* are significantly increased in rats after chronic stress. Data are expressed as means ± SEM (Mann–Whitney, *P <**0.05, **P <**0.01, ***P <**0.001, *n *=**10).

Chronic stress resulted in increased levels of proinflammatory cytokines within fat tissue (Fig. 7A, *n *=**10, *P <**0.05 and **P <**0.01). In addition, VEGF levels were increased both in the circulation and within epididymal fat depots (Fig. 7A set 7, and Fig. 8E, respectively). At the adipocyte level, we have identified a perturbation of an inflammatory‐related network (Fig. 7B). Specifically, gene network analysis revealed a highly statistical gene network (*P*=10^‐33^) to be activated in the stressed adipocytes, having as central nodes, IL‐6, and NF‐*κ*B, which are essential inflammatory genes. Furthermore, as shown in the heatmap representation, central inflammatory genes, such as TNF*α*, MCP‐1, IL‐1*β*, and IL‐6, are upregulated (red color) at the mRNA level in fat depots of chronic‐stressed rats (Fig. 7C). Compared with adipocytes isolated from nonstressed rats, our analysis confirmed the significant increase of TNF*α* IL‐6, IL‐1*β*, and MCP‐1 at the mRNA expression level (Fig. 7D, *P *<**0.01, *P *<**0.05, *P *<**0.05, and *P *<**0.01, respectively, *n *=**10) following stress. Similar increases in inflammatory cytokine levels during stress were also observed in the circulation where in plasma isolated from rats poststress period the levels of IL‐1*β*, IL‐6, IL‐18, TNF*α*, and MIP‐1*α* were significantly higher than in the plasma of control animals (Fig. 8A–D and F, *P <**0.05 and **P <**0.01, *n *=**10).

### Phosphoprotein analysis revealed that stress induces adipocyte intracellular kinase circuits

**Figure 9.**
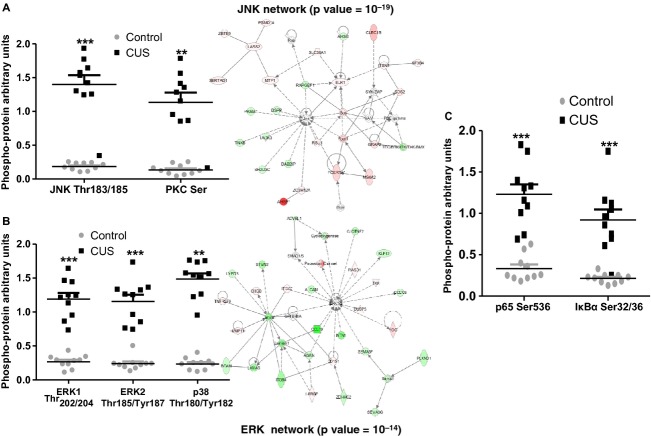
Stress‐induced intracellular signaling pathway activation in rat epididymal adipocytes. Adipocytes were isolated from epididymal fat depots after collagenase digestion and total protein was collected and subjected to multiplex immunoassay using a panel of 13 intracellular signaling phosphor‐kinases. (A) Activation of JNK, PKCs as well as (B) the NF‐*κ*B pathway and (C) MAPK pathways are activated by stress (the networks for JNK and ERK are also identified as among the top activated in our network analyses, sides of A and B) have been shown to adversely affect insulin signaling in different tissues. (Mann–Whitney, **P <**0.01, ***P <**0.001, *n *=**10).

We performed phosphoproteomic analysis, in adipocytes isolated from stressed and control rats to identify the stress‐induced intracellular effectors. We found changes in the activation of several phosphokinases involved in the propagation of a variety of intracellular signaling cascades (Fig. 9, *n *=**10) previously shown to contribute to the development of insulin resistance in different tissues. Stress induced activation of JNK and of total PKC isoforms (Fig. 9A, *P *<**0.001 and *P *<**0.01, respectively, *n *=**10), which are associated with insulin resistance during obesity. In accordance with these data, gene network analysis revealed a JNK‐related gene network (*P*=10^‐19^) to be activated in stressed adipocytes. We also observed that stress induces the activation of ERK1/2, and p38 in rat adipocytes (Fig. 9B, **P <**0.01 and ***P <**0.001, *n *=**10) consistent with our gene network data which revealed an ERK‐related gene network (*P*=10^−14^) to be activated in the stressed rat adipocytes. Finally, data demonstrated activation of the p65 subunit as well as phosphorylation (that leads to degradation) of its inhibitor I*κ*B*α* (Fig. 9C, *P *<**0.001, *n *=**10).

## Discussion

Epidemiologic studies increasingly support an association between anxiety disorders and metabolic disturbances. The relationship is bidirectional and depression appears as a risk factor for the development of diabetes as demonstrated by impaired glucose tolerance in depressed patients (Hennings et al. 2012). However, the neurobiology underlying the co‐occurrence of depression and altered glucose metabolism is poorly understood. While some animal models have been developed to explore the relationship between increased overall adiposity and changes in glucose metabolism (Lamounier‐Zepter et al. 2006; Bose et al. 2009), the contribution of visceral fat in the development of stress‐associated insulin resistance and the physiological and functional changes in adipocytes have not been investigated. Reports investigating the association between change in body weight (Depke et al. 2008; Bartolomucci et al. 2009) and profound metabolic dysregulation with stress (Depke et al. 2008) have shown enormous methodological differences and have mostly focused on hepatic and muscle tissues, leaving the characterization of adipose tissue involvement incomplete and not well understood. Our study provides an in‐depth characterization of the adipose tissue‐associated changes with chronic unpredictable stress and points to specific adipocyte‐related mechanisms that may be responsible for the stress‐associated metabolic phenotype described by us and others.

The model of chronic unpredictable stress used in our study has been extensively characterized as a model of depression in rats. The stressors include, among others, changes in the light/dark cycle as well as changes in the feeding cycle induced by overnight food deprivation. The change in the sleep‐awake pattern (which may affect the locomotor activity) and the change in the feeding pattern (showing increased food consumption in light cycles after an episode of food deprivation) may have a direct effect on insulin sensitivity or glucose metabolism as shown in other studies (Bartness et al. 1989; Gonnissen et al. 2013). In our model, these stressors were combined with others such as social stress (crowding) or rotation or wet bedding, all contributing to generate a sustained unpredictable environment. This combination of stressors presents strong translational value in view of the human experience in which unpredictable changes in sleep, food access, and environment have been shown to have a great impact on anxiety and contribute to changes in metabolism. The effect of stress on abdominal adiposity has been confirmed in nonhuman primates studies in which social stress was associated with increased visceral fat depot, metabolic syndrome, and increased predisposition for cardiovascular diseases and anxiety‐related disorders (Shively et al. 2009).

We show that changes in body composition with chronic unpredictable stress reflect loss of lean mass with a parallel increase in total fat mass (Fig. 2). Interestingly, intraabdominal fat depot masses (linked to metabolic syndrome (Despres and Lemieux 2006; Slentz et al. 2009)) also increase (Fig. 2F and G), potentially contributing to changes in circulating glucose clearing after chronic stress (Fig. 5B). Those changes in body composition were not observed in rats exposed to a single day of stress suggesting the important role of sustained elevated glucocorticoids in the development of the increased visceral adiposity phenotype.

We show that intraabdominal fat depot expansion is mainly due to increased cell numbers rather than cell size (Fig. 4). The physiological status of these new cells is important since the inflammatory conditions present within the fat depot during stress adversely affect preadipocyte differentiation with significant functional consequences (Lehrke and Lazar 2005; Hotamisligil 2006). Our data indicate increased Akt phosphorylation in adipocytes following stress (Fig. 4E). Akt affects multiple opposing pathways depending on extracellular stimuli that regulate its partners. For example, Akt increases cellular proliferation via tuberin inactivation and the subsequent activation of mTOR (Ma and Blenis 2009). The combination of activation of mTOR and its downstream target p70SK6 as well as inhibitory phosphorylation of GSK3*β* in our study (Fig. 4E) suggests that Akt may play a pro‐survival, non‐insulin‐sensitizing role, consistent with increased fat depot sizes with stress. In agreement with this hypothesis, MAPK and NF‐*κ*Β pathway activation (Fig. 9) and the increased levels of VEGF within adipose tissue support an Akt‐induced increased adipocyte survival (Stubbs et al. 2012) promoting adipose tissue remodeling and expansion with stress.

Previous studies demonstrated that impaired adipocyte function and impaired glucose uptake are associated with insulin sensitivity in muscle and liver (Abel et al. 2001). In addition to high circulating NEFA levels (Fig. 5C) which may contribute to reduced glucose clearance with stress by adversely affecting its uptake in liver and muscle (Griffin et al. 1999; Yu et al. 2002), we found increases in circulating proinflammatory cytokines after chronic stress (Fig. 8). It is noteworthy that while some of the cytokines affected by stress (TNF*α*, IL‐6, IL‐1*β*, and MIP‐1*α*) were elevated in both the circulation and fat tissue, levels of IL‐1*α*, MIP3*α*, RANTES, CM‐CSF, and IL‐17 were increased only within the adipose tissue (Fig. 7). These fat‐derived molecules may represent important components of adipose tissue‐associated paracrine regulation of glucose metabolism both via direct effect on insulin signaling cascades and via upregulation of NEFA production by adipocytes. The low levels of circulating insulin (Fig. 5C) in stressed animals deprive them of its antilipolytic effects potentially leading to further increases of NEFA in the blood with all the associated metabolic consequences. This is supported by our observation that treatment with insulin after stress reduces the levels of circulating NEFA back to control levels (Fig. 5D). These data suggest that the effects of stress on insulin resistance in adipocytes may involve the regulation of intracellular signaling mechanisms that alter their glucose uptake capacity do not affect the antilipolytic capacity of insulin.

Our results also indicate that stress induces extensive changes in the transcriptome of adipocytes in rats with major effects on pathways associated with insulin metabolism suggesting adverse regulation of its actions in fat. In agreement with this hypothesis we observed that stress impairs the ability of rat epididymal adipocytes to internalize glucose in response to insulin stimulation (Fig. 6). This response is associated with stress‐dependent activation of intracellular signaling molecules in adipocytes, that either respond to inflammatory signals (p65, p38) or actively phosphorylate IRS‐1 and inhibit insulin signaling (JNK, PKC, ERK1/2) (Fig. 9). Several of these molecules participate in both processes by inducing inhibitory phosphorylation of IRS‐1 in response to proinflammatory extracellular signals and may be responsible for the changes in adipocyte insulin responsiveness observed with stress (Fig. 6). Our data in Fig. 7 suggest the presence of a stress‐induced inflammatory milieu within adipose tissue which can be potentially involved in the activation of insulin resistance‐promoting intracellular signaling cascades in adipocytes. We confirmed the expression of cytokines by adipocytes supporting their potential participation in the promotion of this inflammatory loop. Since these cytokines have been implicated in the induction of insulin resistance in fat (Hotamisligil et al. 1995; Christiansen et al. 2004; Cai et al. 2005), our results support the hypothesis that stress‐induced changes in glucose metabolism are, at least in part, adipocyte related.

Collectively our data suggest significant stress‐related changes in adipose tissue physiology and function that lead to the potentiation of inflammatory responses and/or release of NEFA in the circulation, events associated with impairments in glucose metabolism in humans. Although these changes in adipose tissue are likely coupled with other stress‐induced effects on several other tissues in the body (as shown by the reduced insulin levels in the circulation of the CUS group), our study emphasizes an important contribution of adipocytes in the overall metabolic alterations following stress.

In summary, chronic unpredictable stress is associated with activation of intracellular molecules involved in the inhibition of insulin signaling cascades in adipocytes. These effects are known to be detrimental to insulin sensitivity in other key tissues for the regulation of glucose metabolism (liver and muscle). Coupled to the inflammatory cytokine profiles within fat depots in our stress model, such changes produce a molecular stamp reminiscent of obesity‐like changes in adipocyte function related to insulin sensitivity. However, our evidence suggests pro‐proliferative changes consistent of adipose tissue hyperplasia with stress (similar to those observed in “creeping” fat during Crohn's disease (Desreumaux et al. 1999)) and further studies, characterizing stress‐associated adipocyte physiology and adipose tissue function, are required to effectively assess their importance in disease pathophysiology. Our results highlight the importance of intraabdominal adipocytes as key regulators of the effects of stress on insulin resistance, glucose metabolism, and type II diabetes mellitus.

## Conflict of Interest

None of the authors has any conflict of interest to disclose.
